# The Role of Membrane-Bound Extracellular Vesicles During Co-Stimulation and Conjugation in the Ciliate *Tetrahymena thermophila*

**DOI:** 10.3390/microorganisms13040803

**Published:** 2025-04-01

**Authors:** Eric S. Cole, Oleksandr Dmytrenko, Mark Li, Neetij Krishnan, Josh Thorp, LeeAnn Higgins, Todd Markowski, Garry Morgan, Eileen O’Toole

**Affiliations:** 1 Biology Department, St. Olaf College, Northfield, MN 55057, USA; thorp2@stolaf.edu; 2 Center for Cardiovascular Research, Departmental of Medicine, Cardiovascular Division, Washington University School of Medicine, 660 South Euclid Campus Box 8086, St. Louis, MO 63110, USA; 3 Division of Endocrinology, Diabetes and Metabolism, Beth Israel Deaconess Medical Center and Harvard, Medical School, Howard Hughes Medical Institute, Boston, MA 02115, USA; 4 Department of Pediatrics, Division of Hematology/Oncology, Washington University School of Medicine, St. Louis, MO 63110, USA; 5 Center for Metabolomics and Proteomics, Department of BMBB, University of Minnesota, Minneapolis, MN 55455, USA; 6 Boulder Electron Microcopy Facility, Department of Molecular, Cellular and Developmental Biology, University of Colorado, Boulder, CO 80309, USA; garry.morgan@colorado.edu (G.M.); eileen.otoole@colorado.edu (E.O.)

**Keywords:** ciliates, *Tetrahymena*, conjugation, co-stimulation, ectosomes, EMVs, pheromones

## Abstract

During sexual reproduction, the freshwater ciliate *Tetrahymena thermophila* sheds membrane-bound vesicles into the extracellular environment (cEMVs: ciliary extracellular micro-vesicles). We provide evidence that 100 nm vesicles shed from the cilia of starved cells promote mating between cells of complementary mating types. A proteomic analysis revealed that these EMVs are decorated with mating-type proteins expressed from the MAT locus, proteins that define a cell’s sex (one of seven). Once the mating junction is established between cells, smaller 60 nm vesicles (junction vesicles) appear within the extracellular gap that separates mating partners. Junction vesicles (jEMVs) may play a role in remodeling the mating junction through which gametic pronuclei are exchanged. Evidence is presented demonstrating that cells must be able to internalize extracellular signals via some form of endocytosis in order to trigger conjugation. Finally, an evolutionarily conserved fusogen (Hap2) implicated in pore formation also appears necessary for jEMV processing. This system offers an excellent opportunity for studies on ectosome shedding, intercellular signaling and shed vesicle uptake by macro-pinocytosis, as they relate to sexual reproduction in the ciliate *Tetrahymena thermophila*.

## 1. Introduction

Ciliates are classic ‘protists’ belonging to the Alveolate clade along with Apicomplexans (malaria-like parasites: *Plasmodium*, *Toxoplasma*, and *Monocystis*) and Dinoflagellates (‘red tide’, bio-luminescence, and coral symbionts). Sexual reproduction is well-documented in ciliates and apicomplexans but has been less well-studied among the Dinoflagellates. All three groups exhibit radically different forms of sexual reproduction. The malaria-like parasites spend most of their lives in a haploid condition, briefly forming diploid zygotes through cell fusion. This zygote promptly undergoes meiosis restoring the haploid form [1]. Dinoflagellates also spend most of their lives in a haploid form, briefly fusing to form zygotes, and undergoing a curious, two-step meiosis to restore haploidy [2].

Sexual reproduction among ciliates is unique in several ways [3,4,5]. Ciliates possess two nuclei, a diploid transcriptionally silent micronucleus (Mic) serving as the cell’s germline, and a poly-copy, transcriptionally active macronucleus (Mac) that serves as the cell’s somatic genome [6]. Most of the life cycle is spent with the Mic in a diploid state. *Tetrahymena thermophila* (the subject of this study) exhibits seven different mating types controlled by somatic recombination within the mating-type gene locus [7].

Initiation of conjugation requires nutritional deprivation and the mixing of cells expressing complementary mating types [8]. Following starvation, mixed-mating-type cells undergo a process called ‘co-stimulation’ [9,10] (mediated via brush-by encounters that trigger cell-surface modifications that prepare them for cell adhesion and cell-fusion. Co-stimulation requires a period of physical contact between starved cells of complementary mating types. This conclusion was reached by demonstrating that cultures of cells with complementary mating types separated by a Millipore filter with 8 μm diameter pores failed to provoke co-stimulation through a diffusible substance [11]. Physical contact between mating partners appears to be necessary. Miyake [12] theorized that *Tetrahymena* cells interact through transient direct interactions involving surface ligands and receptors, but this is not the whole story. Direct physical contact, though necessary, is insufficient to bring about pairing. This was demonstrated by the observation that washing co-stimulated cells delays pairing by over an hour and repetitive washings on an hourly basis can prevent conjugation indefinitely [13]. It seems to take time for cells to condition their medium with the necessary soluble factors. Blocking transcription during the period of co-stimulation also prevents pair formation [14,15]. Furthermore, conditioned medium (supernatant collected from co-stimulating cultures) restores mating readiness in washed, co-stimulated cells [13,16,17]. The Wolfe laboratory demonstrated that there is a cell-free factor necessary for co-stimulation. It is large, retained by dialysis, and heat stable. Enigmatically, materials released by either mating type into the starvation medium alone can support co-stimulation when cells are mixed [17]. Cell-free material is also necessary for the process of cell adhesion or pair formation [18]. Wolfe named this material ‘FAC’ or ‘factor active in conjugation’ [19]. All these observations suggest complex signaling between cells of complementary mating types with a role for both direct cell–cell contact (brush-by encounters), and secretion of some large, extracellular material into the surrounding medium, in order for mating partners to form a physical bond and initiate conjugal development (reviewed by Cole [20]). Work in our lab suggests that co-stimulation involves the shedding of extracellular micro-vesicles from the ciliary tips expressing the mating-type proteins on their surfaces. We refer to these as ciliary EMVs or cEMVs to distinguish them from another class of EMVs that appear at the non-ciliated membrane junction (jEMVs).

Cell signaling through the shedding and uptake of extracellular micro-vesicles (EMVs) has emerged as a mechanism that can prepare cells (or organisms) of different sexes to initiate sexual reproduction or conjugation. This was first observed in the unicellular algae *Chlamydomonas reinhardtii* [21,22]. In *Chlamydomonas*, the ‘plus’ algal gametes shed vesicles carrying a mating type factor capable of binding to and stimulating ‘minus’ cells in preparation for mating [23]. Similar results have been found in the metazoan (roundworm) *Caenorhabditis elegans*. In the roundworm, extracellular vesicles are released by the hermaphrodite into the environment from ciliated sensory neurons. These vesicles have the capacity to modulate mating behaviors in males reversing their locomotion, and driving tail-curling maneuvers in preparation for conjugation [24].

**Figure 1:**
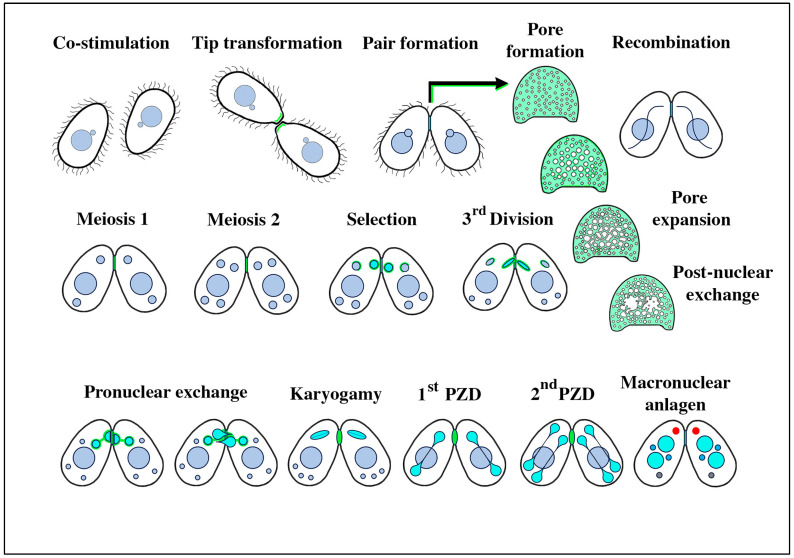
Stages of conjugation in the ciliate *Tetrahymena thermophila*. The mating junctions (pale green) are enlarged and en face to highlight details of pore formation and expansion, and the disruption following pronuclear exchange that must be repaired to restore cellular integrity. Turquoise nuclei represent the haploid, the ‘selected’ micronucleus within each partner just following meiosis, and their mitotic products (the migratory and stationary pronuclei) during nuclear exchange and fertilization. Following fertilization, the postzygotic nuclei are also turquoise. During macronuclear anlagen formation (differentiation of the newly recombinant genome into a polyploid pair of macronuclei), one can observe a non-membrane-bound organelle in the anterior cytoplasm (shown in red) named the ‘conjusome’ [28].

In *Tetrahymena*, early responses to co-stimulation include two physical changes. First, cell-surface conA-receptors accumulate at the anterior tip of the cell (detectable by fluorescent conA ligands, as illustrated in green in Figure 1). Second, the anterior tip of the cell establishes a cilium-bare patch that will form the future adhesion platform [13,25]. Proteins involved in triggering co-stimulation were first identified in *Tetrahymena* [7,26] and more recently, in *Paramecium* [26]. In *Tetrahymena*, each cell expresses two mating-type proteins (MTA and MTB) that are unique to its individual mating type. Cells from which MTA has been deleted, but still in possession of MTB, can trigger conA receptor aggregation in a partner’s mating tip, but cannot trigger assembly of a functional adhesion platform [27].

Both MTA and MTB proteins are required for tip transformation [27]. MT proteins appear to form a complex with one another and with six other components (MRC1-6), forming a mating-type recognition complex (MTRC). MRC4 and MRC5 are predicted to be Ca^2+^ translocators implicating calcium signaling during co-stimulation. MT proteins also co-express (and immune precipitate) with three other proteins, CDK19, CYC9, and CIP1, which constitute a cyclin-dependent kinase complex that is localized to the cell tip and pairing junction and is essential for mating [29,30]. MT proteins also co-express (and are associated with) AKM3, a novel K^+^ channel. These studies reveal that mating-type recognition involves a rich multi-protein complex involved in both self and non-self-recognition during co-stimulation. Of particular significance to our investigation, these studies located MTA and MTB on the cell surface where they co-localize, but not on ciliary membranes.

Following 20–40 min of co-stimulation at 30C, *Tetrahymena* begin to adhere to one another at the anterior mating junction [9]. Cells join at this adhesion zone separated from one another by a uniform intercellular gap of about 50 nm. Such cell associations are referred to as ‘loose’ pairs in that they can be easily disrupted by mechanical agitation. Loose pairs are frequently missed in studies that first centrifuge mating cells or apply a coverslip before counting. ‘Tight pairs’ form about 1.5–2 h after cells are mixed. These persist against mild mechanical agitation, presumably because of the structural support established by hundreds of independent membrane fusion events all occurring within the mating junction [31]. Little is known about the molecules involved in the cell’s initial adhesion to a partner, though recent work by Yan et al. [27] suggests that the mating-type recognition complex (MTRC) plays a role in triggering cell recognition and mediating cell adhesion.

**Figure 2:**
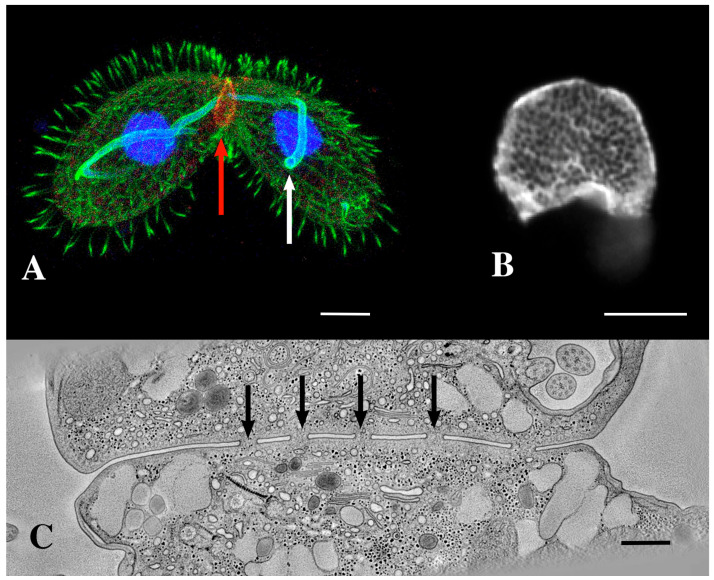
(**A**) A pair of mating *Tetrahymena* cells decorated with anti-acetylated tubulin (green) and expressing LF4B-GFP, an RCK Kinase involved in determining the cilia length that decorates the mating junction, analyzed by immunofluorescence using anti-GFP antibodies (red) 12G10 anti-α-tubulin (green) and DAPI (blue) (from Jiang et al., 2019 [33]) Scale bar = 10 µM. The white arrow indicates the crescent elongation of the pre-meiotic chromosomes. The red arrow indicates the mating junction. (Photo courtesy of Jacek Gaertig). (**B**) An en-face view of an isolated mating junction decorated with fenestrin:GFP (photo courtesy of Benjamin Reister) Scale bar = 5 µM. One can see over a hundred fusion pores. (**C**) A transmission electron micrograph cross section of the mating junction. Arrows indicate fusion pores in profile. (Image taken as a single frame from a 3-D tomogram first published by Cole, et al. [34]. Scale bar = 400 nm.

Mating pairs do not fuse to form a single, diploid, zygotic product. Rather, hundreds of transient, focal cell fusions appear in a limited, specialized region of the cortical cytoplasm anterior to the mouth (or oral apparatus, OA) (Figure 2B,C) (see also Wolfe [25]). Later, after nuclear exchange, karyogamy, and recombinant nuclear differentiation, cells restore their individual integrity via an elaborate process of membrane remodeling [32].

Cell fusion is mediated by the *Tetrahymena* homolog of the Hap2/GCS1 fusogen [31,35,36,37]. It is of considerable interest that this male-gamete-specific fusogen is conserved between ciliates, apicomplexans and amoeba, as well as algae, higher plants and even primitive animals including cnidarians and some insects [38]. This argues for the ancient origin of a common mechanism mediating gamete fusion across the entire eukaryote lineage [39]. It is also notable that the Hap2/GCS1 protein appears to be structurally and functionally homologous to class II membrane fusogens identified in viruses such as Zika and Dengue [35]. Each fusion event creates a cytoplasmic bridge between mating partners, and each pair becomes joined by over a hundred of these membrane pores.

**Figure 3:**
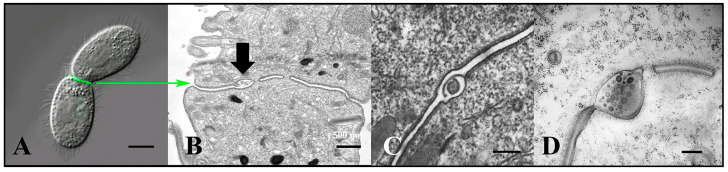
Extracellular vesicles (jEMVs) being shed into the mating junction. (**A**) Intact pair with DIC and fluorescence microscopy highlighting conA label. (Scale bar = 10 µM). (**B**) TEM image of mating junction. Arrow highlights extracellular pocket within mating junction with jEMVs (scale bar = 500 nm). (**C**) Close-up TEM of wild-type mating junction capturing what appears to be a macro-pinocytotic membrane fold enveloping a jEMV (scale bar = 250 nm) (Cole et al., 2015 [34]). (**D**) A *bcd1 × bcd1* mutant mating junction showing excess accumulation of jEMVs (scale bar = 250 nm).

During meiosis, the margins of the pores expand, and their expansion may cause them to coalesce with other expanding pores, creating a tubular membrane reticulum or ‘curtain’ that separates the cytoplasm of the two mating partners (Figure 1) [3,34]. During this period, pores expand from 100 to 200 nm in diameter to 320 to 450 nm in diameter [25]. Also, during this period, we observe membrane-bound vesicles being shed into the inter-cellular space that comprises the mating junction (Figure 3B) [34]. These are the junction-derived extracellular micro-vesicles or jEMVs, distinct from the ciliary EMVs (cEMVs) described earlier. It seems there are two examples of membrane vesicle production during *Tetrahymena* conjugation.

Once vesicles are broadcast, the question becomes the following: ‘do these vesicles interact with the recipient and how are they received?’ Multiple routes of EMV uptake have been described [40]. In *Tetrahymena*, at the mating junction, we have observed what appear to be membrane folds enveloping and internalizing shed vesicles (Figure 3C) [34]. We hypothesize that membrane vesicles shed into the extracellular space of the mating junction are subsequently internalized through macro-pinocytosis.

Here, we report observations regarding ciliary EMVs and their potential role in co-stimulation leading to pair formation, and junction EMVs and their potential role in membrane remodeling and miRNA activity at the nuclear exchange junction, following meiosis. We hope this serves as a catalyst for future explorations.

## 2. Methods

### 2.1. Cell Culture

Cells were grown at 30C in ‘NEFF’ medium (0.25% proteose peptone, 0.25% dextrose, 0.5% yeast extract, 0.009% ferric EDTA). Strains included CU428 and SB1969, as well as strains of functional heterokaryons CU427 and CU428 in which the *HAP2* gene had been deleted [31]. All cell lines were from the *Tetrahymena* Stock center, Cornell University. Cells of complementary mating types were grown to densities of 2 × 10^5^ to 8 × 10^5^ cells/mL. Cultures were then centrifuged and transferred to 10 mM Tris at pH 7.4 for overnight starvation (two rinses). After 12 to 18 h of starvation, mating types were mixed at equal densities (in the range from 5 × 10^5^ to 8 × 10^5^ cells/mL). At appropriate times after mixing, samples were harvested and fixed for transmission electron microscopy (TEM).

### 2.2. Pairing Assay

Cells of complementary mating types were starved and mixed at time = zero. EMVs harvested from mixed, wild-type cells 40 min into co-stimulation were then applied to one dish, while EMV buffer (10 mM TRIS, pH 7.4) was added to the control cells in another. Every 10–15 min, a sample was gently pipetted to a slide (very gentle cover-slip application using clay feet on corners to prevent mechanical disruption), and cells were scored as either single or paired using an Olympus CH2 microscope at 100× magnification. Pairs were counted as single objects. Graphs were plotted as the % “objects” identified as pairs over the total # “objects”.

### 2.3. Preparation for Thin-Section TEM

Mating cells (pairing cultures confirmed by light microscopy) were sampled at various times and processed for electron microscopy as previously described [41].

### 2.4. Freeze-Substitution TEM

Cultures with mating pairs of *Tetrahymena* were prepared for transmission electron microscopy as described in [41,42]. Briefly, cells were harvested by centrifugation into a cryoprotectant solution consisting of 15% dextran (9–11 KD, Sigma) and 5% BSA in 10 mM Tris and frozen in a Wohlwend Compact 02 High Pressure Freezer. Samples were freeze-substituted in 2% osmium tetroxide, 0.1% uranyl acetate in acetone and embedded in Spurr’s epoxy resin. Serial thin sections were imaged using a Philips CM100 TEM.

### 2.5. Electron Tomography

Electron tomography of *Tetrahymena* was performed as described [41]. Briefly, serial thick (250 nm) sections were collected onto formvar-coated slot grids and stained with 2% uranyl acetate and Reynold’s lead citrate. Then, 15 nm gold particles were affixed to the sections for use as alignment markers. Tomography was carried out using a TECHNAI F20 or F30 electron microscope (FEI Corp., Hillsboro, OR, USA) using the SerialEM image acquisition program [43]. Dual-axis tilt series were collected every 1° over a +/−60° range using a Gatan 2 k × 2 k CCD camera at a pixel size of 1.2–1.5 nm. The tilt series were aligned and tomograms computed and modeled using the IMOD software package [44].

### 2.6. Negative Staining

Cells of complementary mating types were fixed at 40 min after mixing in 2.5% glutaraldehyde. Then, 10 µL of fixed cells/solution were transferred onto a carbon-coated mesh TEM grid (at room temp), which was incubated for 1 min. Cell solution was blotted off, the grid was washed in H_2_O 10 s and then blotted off. UranyLess staining solution was added for about 30 s and then blotted off. The negative stained sample was imaged on a Tecnai T12 Spirit Biotwin 100 kV TEM.

### 2.7. TEM on Pelleted EMVs

Vesicles harvested by differential centrifugation were applied to carbon-coated copper grids and stained in 0.4% uranyl acetate and 2% methyl cellulose as described by Hacker et al. [44]. This method preserves the three-dimensional structure of micro-vesicles.

### 2.8. EMV Isolation by Differential Ultra-Centrifugation

Our protocol was taken from the work of Thery et al. [45] (Supplementary File S1).

### 2.9. EMV Size Analysis

Electron micrographs of pelleted EMVs (isolated from the extracellular medium) or junctional EMVs (imaged in situ at the mating junction) were used to measure vesicle diameters.

### 2.10. Mass Spectrometry of EMV Contents

#### 2.10.1. Protein Extraction

All samples were prepared as follows: extracellular microvesicles were reconstituted with 30 µL of extraction buffer [7 M urea, 2 M thiourea, 0.4 M triethylammonium bicarbonate (TEAB) pH 8.5, 20% acetonitrile and 4 mM tris(2-carboxyethyl) phosphine (TCEP)]. The samples were vortexed briefly, and then each sample was transferred to a Pressure Cycling Technology (PCT) tube with a micropestle cap to be used in the Barocycler NEP2320 (Pressure Biosciences, Inc., South Easton, MA, USA). Pressure cycled between 35 kpsi for 20 s. and 0 kpsi for 10 s. for 60 cycles at 37 °C. The PCT tube was uncapped and 200 mM methyl methanethiosulfonate (MMTS) was added to a final concentration of 8 mM MMTS, recapped, inverted several times and incubated for 15 min at room temperature. The samples were transferred to a new 1.5 mL microfuge Eppendorf Protein LoBind tube. Two aliquots for each sample were taken for protein concentration determination by Bradford assay.

#### 2.10.2. In-Solution Proteolytic Digestion

Aliquots of each sample were transferred to a new 1.5 mL microfuge tube and brought to the same volume with protein extraction buffer plus 8 mM MMTS. All samples were diluted four-fold with ultra-pure water and trypsin (Promega, Madison, WI, USA) was added in a 1:40 ratio of trypsin to total protein. Samples were incubated overnight for 16 h at 37 °C. After incubation, they were frozen at −80 °C for 0.5 h and dried in a vacuum centrifuge. Each sample was cleaned with a 1 CC Oasis MCX solid-phase extraction cartridge (Waters Corporation, Milford, MA, USA) and the eluate was dried in vacuo.

#### 2.10.3. Mass Spectrometry

We performed a micro-scale clean-up procedure on the mixture of trypsin peptides (approximately 4 μg material was digested) using the Stage Tip protocol with SDB-XC reverse-phase material from 3M (St. Paul, MN, USA) according to the published protocol [REF: PMID 12585499] and dried the peptide mixture in vacuo. We reconstituted the pellet in ten microliters of 95:5, water: acetonitrile, 0.1% formic acid, adjusted the pH to 2 and analyzed one tenth of the sample by capillary LC-MS on an Orbitrap Velos (Thermo Fisher Scientific, Waltham, MA, USA) mass spectrometer system with higher-energy collision-induced dissociation (HCD) activation as previously described [REF: PMID 23148228] with slight modifications. The LC modifications were as follows: 100 micrometer inner diameter C18 column, column length 14 cm, gradient elution profile 2–8% B Solvent (where A Solvent was 98:2:0.01, water: acetonitrile: formic acid and B Solvent, 98:2:0.01, acetonitrile: water: formic acid) in 2 min, 8–35% B in 67 min, 35–90% in 1 min and 90% B for 7 min at a flowrate of 300 nL/min. We altered the following mass spectrometer acquisition settings: no lock mass was employed, MS1 range was 360–1800 *m*/*z*, dynamic exclusion maximum number of values was 200, duration was 30 s and exclusion mass tolerance was ±10 ppm. We re-analyzed the sample with the identical injection amount and LC parameters but performed CID (collision-induced dissociation) activation instead of HCD. The CID-specific MS parameters were as follows: maximum injection time 100 ms and ion trap AGC 1 × 10^4^.

#### 2.10.4. Database Search

We used PEAKS Studio 8.5 (Bioinformatics Solutions Inc., Waterloo, ON, Canada) software for database searches with proteins from *Tetrahymena thermophila* (taxonomy ID 5911) downloaded on 1 September 2017 from NCBI Reference Sequence repository merged with the common lab contaminants database from https://www.thegpm.org/crap/ (accessed on 1 March 2015), and four *Tetrahymena* mating proteins (NCBI Database: https://www.ncbi.nlm.nih.gov (accessed on 1 March 2015), gi|449785212, gi|449785211, gi|449785194, gi|449785193). We set the PEAKS search parameters to precursor mass error tolerance 50.0 ppm; fragment mass error tolerance 0.1 Da; precursor mass search-type monoisotopic; enzyme trypsin, max missed cleave site 1, non-specific cleavage none; variable modifications: methionine oxidation and di-oxidation, asparagine and glutamine deamidation, oxidation of histidine and tryptophan, pyroglutamic acid from N-terminal peptide glutamine; protein N-acetylation; fixed modification beta-methylthiolation of cysteine, maximum variable modifications per peptide 3; false discovery rate calculation; spectra merge options: 0.2 min within 15.0 ppm mass window; charge correction on for charge states 2–9; spectral filter quality > 0.65. We set the false discovery rate threshold for peptides and proteins at ≤1% in the Summary page.

### 2.11. RNA Isolation and Size Analysis for cEMVs from Wild-Type Co-Stimulated Cells

Isolated EMVs were shipped to the University of Minnesota on dry ice and frozen at −80 °C upon receipt. EMV RNA was extracted using the Qiagen exoRNeasy Serum/Plasma kit, bypassing the isolation steps and beginning with QIAzol lysis (Step 6). Extracted RNA was evaluated by RiboGreen (ThermoFisher, Waltham, MA, USA) quantification and Nanodrop. Further analysis of the size profiles was conducted using an Agilent Bioanalyzer 2100 with both Small RNA and RNA 6000 Pico kits. Then, 10 ng of EMV-derived RNA was used as input into the Clontech SMARTer smRNA-Seq Kit (Takara, Shiga, Japan). The prepared library was size-selected using AMPureXP beads (Beckman Coulter, Brea, CA, USA) in order to enrich inserts < 150 bp. The size-selected library was sequenced on a single lane of Illumina HiSeq 2500 V4 in single-read mode for 50 cycles.

### 2.12. RNA Isolation and Size Analysis for jEMVs from HAP2D 4 h Mating Pairs

Samples were prepared according to the Illumina small RNA protocol, indexed, pooled, and sequenced on an Illumina HiSeq 2500 rapid run instrument with 1 × 50 bp reads. Basecalls and demultiplexing were performed with Illumina’s bcl2fastq software and a custom python demultiplexing program with a maximum of one mismatch in the indexing read. RNA-seq reads were then aligned to the Ensembl release 31 Tetrahymena thermopile JCVI-TTA1-2.2 assembly with STAR version 2.0.4b [46]. Gene counts were derived from the number of uniquely aligned unambiguous reads by Subread: featureCount version 1.4.5 [47]. Isoform expression of known Ensembl transcripts was estimated with Sailfish version 0.6.13 [48]. Sequencing performance was assessed for the total number of aligned reads, total number of uniquely aligned reads, and features detected. The ribosomal fraction, known junction saturation, and read distribution over known gene models were quantified with RSeQC version 2.3 [49].

### 2.13. Dynasore Treatment for Pairing Assay

Endocytosis was blocked by exposing mixed mating cells to 70 μM Dynasore. A 70 mM stock solution was prepared in DMSO, and diluted 1:1000 into the mating cell mix (10 μL per 10 mL). For controls, 10 μL of DMSO alone was added. Pairs were assessed as described earlier.

## 3. Results

### 3.1. Membrane-Bound Vesicles Are Shed from Cilia During Co-Stimulation and into the Mating Junction During Conjugation and Membrane Remodeling

**Figure 4:**
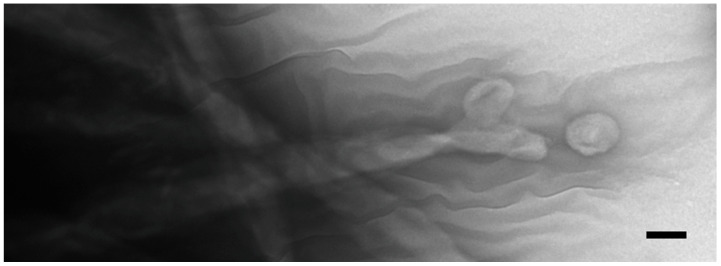
Negative-stained cilia from an intact, starved, co-stimulating *Tetrahymena* cell showing what appears to be concave vesicles shedding from their tips. Scale bar = 100 nm.

When intact co-stimulated cells are fixed and prepared for negative staining, extracellular vesicles appear associated with the ciliary membranes (Figure 4). These images suggest that ciliary membrane shedding occurs during co-stimulation. We refer to these as ‘ciliary extracellular micro-vesicles’ (cEMVs) to distinguish them from vesicles shed from the plasma membrane at the mating junction (‘junction extracellular micro-vesicles’, or jEMVs, as shown below).

**Figure 5:**
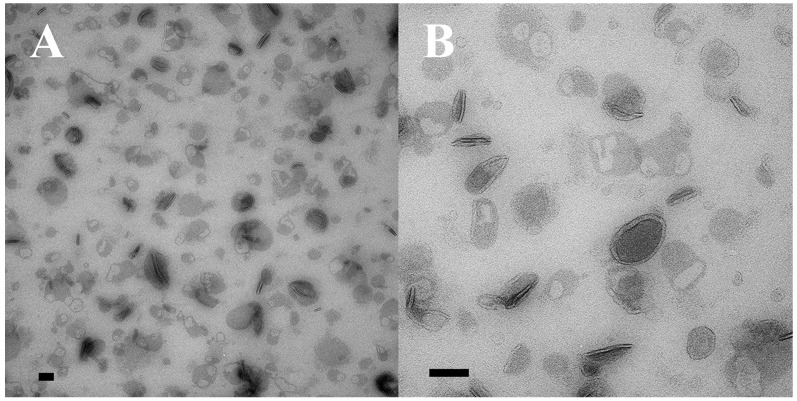
Two samples of ciliary EMVs (cEMVs) collected by differential ultra-centrifugation from co-stimulating *Tetrahymena* cells and prepared for TEM using special methyl-cellulose technique to protect their 3D structure. (**A**) Low mag image, (**B**) High mag image. Scale bars = 100 nm.

**Figure 6:**
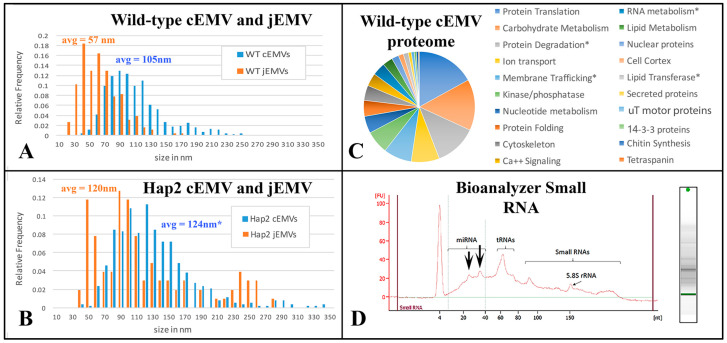
Analysis of both cEMVs and jEMVs collected from mutant and wild-type *Tetrahymena* during co-stimulation and mating. (**A**,**B**) Distribution plots of shed vesicle diameters measured from TEM images of wild-type and *HAP2Δ* mutant matings. Blue cEMVs (ciliary EMVs) were measured from material collected by differential ultra-centrifugation from 4 h mating cells of either (**A**) wild-type partners or (**B**) *HAP2Δ* deletion partners. For the *HAP2Δ* matings, the cEMVs are no doubt contaminated with jEMVs, (junction vesicles) since mating pairs fall apart during centrifugation, releasing vesicles from the mating junction and into the supernatant. This is far less likely from the tightly bound wild-type partners. Junction EMVs (jEMVs) were measured in situ from thin sections of 4 h mating pairs. jEMVs imaged from *HAP2Δ* mating junctions exhibit twice the average size of wild-type jEMVs with significant variance. (**C**) Proteomic analysis of shed vesicles (cEMVs) collected 4 h into mating. This diagram shows proteins clustered by functional annotation. The complete list appears in the Supplementary Materials. (**D**) Spectrophotmetric analysis of small RNAs present in shed vesicles. Note, these EMVs were collected from disrupted *HAP2Δ* matings taking advantage of the mutant pair fragility. Centrifugation disrupts pairing, liberating the jEMVs into the medium for differential centrifugation. This also means we are not analyzing wild-type jEMVs. Asterisks indicate proteins of special interest described in the text.

The appearance of vesicles budding from the ciliary membranes raised the possibility of harvesting them for analysis. We collected cEMVs from mating cell cultures 2 h after mixing. Typically, 700 mL of mixed, mating cells at a density of 600,000 cells/mL yielded a 30 µL pellet of cEMVs. This material was negatively stained and examined with TEM. Images of this material appear in Figure 5. What is notable is the regularity of the material. There is very little material that could be identified as cellular debris. The abundant membrane-bound vesicles have a characteristic flattened disc-shaped appearance. Vesicles isolated in this way (ciliary EMVs or cEMVs) were measured and compared with EMVs photographed in situ within the mating junction (jEMVs, Figure 3B–D). These data appear in Figure 6. The mean diameter for wild-type cEMVs was 105 nm (N = 520, std = 38 nm). EMVs trapped between cells within the mating junction (jEMVs) were smaller, with a mean diameter of 57 nm (N = 256, std = 26 nm).

These results suggest that two distinct classes of vesicles are being shed from mating *Tetrahymena*, i.e., larger ones (105 nm) from the cilia, and smaller ones (57 nm) from the un-ciliated plasma membrane that forms the mating junction. We have not determined whether active vesicles are shed continuously, during vegetative growth or during starvation prior to co-stimulation and mating.

### 3.2. The Wild-Type cEMV Proteome and the HAP2Δ jEMV Transcriptome

EMVs were harvested from 4 h co-stimulating cells and their proteins were analyzed by mass spectrometry (Supplementary File S2). In total, 483 proteins were identified. Of these, 123 were characterized as ‘hypothetical’ proteins. The remaining 361 could be annotated as orthologs to known proteins or protein functions. These are categorized in Figure 6C. Protein constituents from shed vesicles were similar to the micro-vesicle constituents published from other organisms (*Drosophila*, mammal, plant; [50,51,52]. The most abundant proteins were associated with protein translation (17%) and carbohydrate metabolism (15%). The third most abundant category involved protein ubiquitination and degradation via the proteasome as well as a variety of proteases (13%). These three categories account for nearly half of the identifiable protein functions. The next most populous protein categories were proteins involved in transporting ions across membranes, especially proton transport (8%), and membrane vesicle trafficking (8%). Seven percent of the protein constituents were involved in regulation of phosphorylation and de-phosphorylation (kinases and phosphatases). Three categories each constitute 5% of the proteome: proteins associated with mono-nucleotides, calcium regulation and signaling, and protein folding (chaperonins). Following these (at 3%) were an interesting class of proteins associated with miRNA. These included TWI1 and TWI2 (orthologues of the argonaute proteins involved in RNAi), and several DEAD/DEAH box helicases. Also at 3%, there were proteins associated with the cytoskeleton (especially notable: actin and actin-binding proteins). At 2%, there were proteins involved in lipid metabolism, nuclear proteins (including histones and transcription factors), lipid transferases (flippase and scramblase proteins) and cortical cytoskeleton proteins.

At 1% or less, there were secreted proteins, microtubule motors, 14-3-3 proteins, chitin synthetase enzymes and tetraspanin. It should be noted that 14-3-3 proteins and tetraspanins are characteristic of shed vesicles [52,53,54,55]. Another 15 proteins were identified as single representatives and are listed in Supplementary File S2.

It should be noted that the abundance of proteins within a category does not necessarily reflect their significance. Metabolic enzymes may be enclosed within shed vesicles as a by-product of non-specific cytoplasmic sampling. Single-copy gene products, on the other hand, might play a major, albeit solo, role in vesicle formation or signaling. One set of proteins that did not appear immediately in our proteomic survey included the mating-type proteins. In this experiment, we mated cells of mating type II and mating type VII. An idiosyncrasy of the *Tetrahymena* genome database is that the genome was published for a cell with mating type = VI. Mating type in *Tetrahymena* is determined by a process of DNA rearrangement, and gene excision. Of the six possible mating-type alleles, five gene are physically eliminated [7]. Consequently, the reference genome (against which we searched for matches to our proteomic peptides) failed to identify these significant proteins. When the mass spec data were re-screened using MT VII and MT II protein sequences, both MTA7 and MTB7 were identified, as well as MTB2. It is likely that MTA2 was also present but not detected in this round of mass spectrometric analysis. Hence, the ciliary vesicles shed during co-stimulation also carry the mating-type proteins.

### 3.3. EMVs Harvested from Both Co-Stimulating Cells and Mating Meiotic Cells Are Rich in RNA

Using a nano-drop analyzer, we determined that EMVs collected 40 min after mixing from co-stimulating wild-type cells possessed RNA. A 30 µL pellet resuspended to a final volume of 150 µL exhibited approximately 67 ng RNA/µL. RNA was extracted from pelleted EMVs and analyzed for size distribution. RNA isolated from shed micro-vesicles appeared to be rich in small and micro-RNAs (Figure 6D). Two spikes appear at approximately 24 and 35 nt (see arrows, Figure 6D). These sizes correspond in size to two classes of small RNAs previously characterized for *Tetrahymena* [56,57].

In an attempt to analyze jEMVs, RNA was also harvested 4 h after cell mixing from *HAP2Δ* pairs that were engaged in meiosis. This experiment on meiosis-stage vesicle contents requires some explanation. We performed this analysis early in our investigation, at a time when we did not know about ciliary EMVs, and only knew about junction EMVs. We anticipated difficulty in harvesting vesicles from the narrow gap that separates tight-mating pairs. We had noticed that *HAP2Δ* mutants produce fragile pairs, yet shed impressive micro-vesicles into the mating junction. This pair fragility is due to their failure to undergo membrane fusion. *HAP2Δ* mutant pairs fall apart during even mild centrifugation, yet their development (including meiosis) proceeds on schedule [31]. *HAP2Δ* mutant pairs also exhibit unusually large jEMVs, as shown in Figure 6B, though most fall in a size range that overlaps wild-type vesicles. For these reasons, we harvested vesicles for meiosis-stage RNA analysis using (*HAP2Δ* × *HAP2Δ*) mutant pairings hoping to release jEMVs from where they were secreted and ‘trapped’ within the cell–cell junction. Vesicles harvested in this manner likely include both cEMVs shed by cilia (and likely to be in greater abundance as they have accumulated throughout the starvation and co-stimulation periods), and jEMVs released from the mating junctions (and probably in far less abundance). RNA analysis revealed short RNA species (under 50 nucleotides) that were mapped to 24,432 genes in the *Tetrahymena* genome.

### 3.4. EMVs Are Active in Conjugation

**Figure 7:**
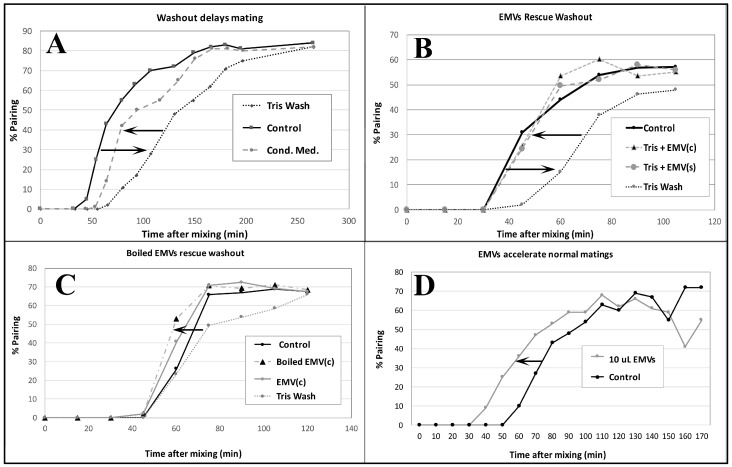
Pairing assays demonstrate the ‘mating activity’ present in conditioned medium collected from starved single mating-type culture (EMVs) and from mating co-stimulating cultures (EMVc). (**A**) Demonstration that washing cells in fresh starvation medium delays pairing by up to an hour. Pairing dynamics are restored by adding back conditioned medium following centrifugation. (**B**) Shed vesicles collected from either starved cells of a single mating type, EMV(s) or from cultures 40 min into co-stimulation EMV(c) can restore pairing when added to fresh, unconditioned starvation medium. (**C**) Shed vesicles that have been briefly boiled can restore pairing delayed by washing out. (**D**) Shed vesicles collected from 40 min co-stimulating cells accelerate pairing in cells mating in normal ‘conditioned’ medium. Pairing assays were performed by gently sampling cells from a mating mixture under the microscope, and counting the number of ‘objects’ (out of a sample of 100) representing pairs vs. single, unmated cells. (# pairs/total # objects) × 100 = % pairs.

Co-stimulated cells washed in fresh 10 mM TRIS buffer (the normal starvation medium) exhibit delayed pairing (Figure 7A). When ‘washed’ co-stimulating cells are resuspended in ‘conditioned’ TRIS medium, pairing is largely restored. Harvested EMVs added to fresh TRIS buffer restore pairing to control levels (Figure 7B). EMVs harvested from starved cells of one mating type {EMV(s)}, or co-stimulating cells {EMV(c)} show equivalent recovery of pairing from TRIS-washed co-stimulating cells of two mating types (Figure 7B).

EMVs boiled for three minutes retain their co-stimulation activity (Figure 7C). One new discovery not observed in the Wolfe lab was that EMVs added to un-washed cells at the time of mixing actually accelerated pairing over control cells by up to 20 min (Figure 7D). These findings demonstrate that starved cells shed material into their medium that exhibits mating activity.

### 3.5. Endocytosis May Be Necessary for Pairing in Tetrahymena

**Figure 8:**
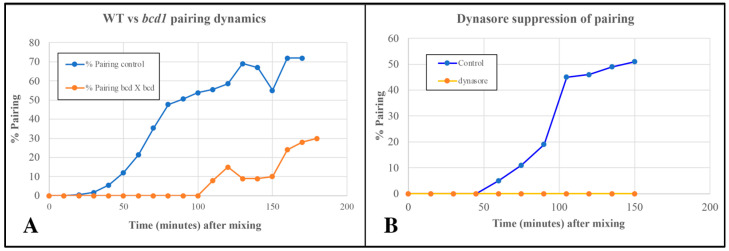
(**A**) Delayed pairing in matings involving cells homozygous for the *bcd*1 mutant defective in endocytosis. (**B**) Inhibition of pairing in wild-type cells exposed to 70 µM Dynasore (endocytosis inhibitor).

The pattern mutant, *bcd*1, is defective in several forms of endocytosis including pinocytosis (from parasomal sacs located at the base of each cilium), and phagocytosis occurring at the oral apparatus [20]. Cells homozygous for the *bcd*1 mutation also show a delay in pairing in the standard TRIS buffer (Figure 8).

Cells treated with 70 µM Dynasore (a known inhibitor of dynamin involved in endocytosis) showed complete suppression of pair formation (Figure 8B). Together, these observations led us to suspect that shed vesicles (that trigger or accelerate pair formation) may be taken up and internalized by some form of endocytosis to bring about co-stimulation leading to pair formation.

### 3.6. EMVs Become Enriched in the ‘Ciliary Pockets’ in bcd1 Mutants Undergoing Conjugation

**Figure 9:**
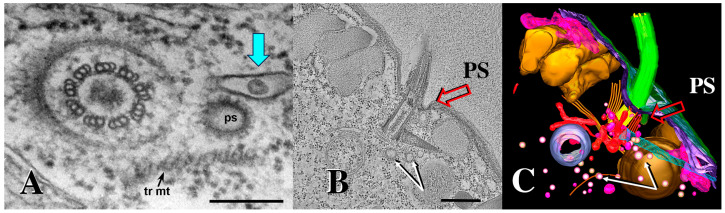
A 3-D electron tomogram of a cilium from a co-stimulated cell with its ciliary pocket and parasomal sac. (**A**) TEM image of wildtype cilium, ciliary pocket and parasomal sac (ps) (from Richard Allen image archive at the University of Hawaii, Manoa. (https://www6.pbrc.hawaii.edu/allen/ch18/, accessed on 1 March 2015). Note: blue arrow indicates previously unidentified vesicle in a membrane pocket within the ciliary pocket. Scale bar = 200 nm. (**B**) A section of a tomogram from a wild-type cell highlighting the cilium, parasomal sac (ps) and clathrin-coated vesicles (white arrows). Scale bar = 400 nm. (**C**) A tomographic slice from (**B**) highlighting parasomal sac and abundant coated vesicles (golden vesicles, white arrows). Note the absence of extracellular micro-vesicles.

We examined cells during co-stimulation and pairing using 3D electron tomography. Normal pinocytotic figures (parasomal sacs, PS) have been observed at the base of every cilium in the wild-type cells (Figure 9).

**Figure 10:**
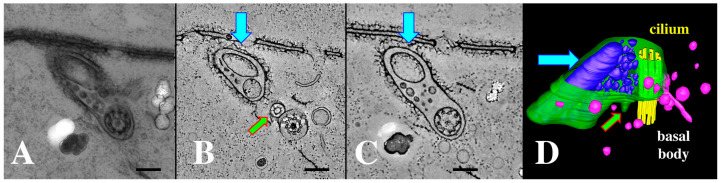
(**A**) cilium and its ciliary pocket from a mating *bcd1* mutant cell with vesicles of varying sizes, filling the ciliary pocket. (**A**) Thick section view used for tomography in (**B**–**D**). (**B**) A tomographic slice of the thick section in (**A**) showing the clathrin-coated parasomal sac (green arrow) adjacent to the cilium. (**C**) A tomographic slice from deeper in the volume showing the continuity of the vesicle-filled pocket with the ciliary pocket (blue arrow highlights a massive EMV). (**D**) The same cytoplasmic volume modeled to reveal the parasomal sac (green arrow), and extracellular vesicles in a variety of sizes (blue) occupying an enlarged ciliary pocket (turquoise arrow). Note the absence of coated vesicles issuing from the parasomal sac (pink vesicles are endosomes without clathrin coats). Scale bars = 250 nm.

While examining archival images of *Tetrahymena* TEMs from the Richard Allen collection (https://www6.pbrc.hawaii.edu/allen/ch18/, accessed on 1 March 2015), we noticed what appears to be a solitary vesicle, distal to the parasomal sac (the site of pinocytosis), and potentially captured in a membrane depression within the ciliary pocket (Figure 9A, turquoise arrow). This formerly unrecognized feature may be significant in light of images collected from mating *bcd*1 mutant *Tetrahymena* (Figure 10). In the mutant, excess accumulation of extracellular vesicles was observed at the base of *bcd*1 mutant cilia, and within enlarged pockets at the base of the cilium, similar to the singular vesicle seen in the wild-type view (Figure 10). These results suggest that extracellular materials (ectosomes) are being internalized by some form of endocytosis within the ciliary pocket, in order to complete co-stimulation and initiate pairing, a process that is disrupted in the *bcd*1 mutant.

### 3.7. Tetrahymena Shed Membrane Bound Vesicles Directly into the Mating Junction Forming ‘Junction EMVs’ (jEMVs) That May Then Be Internalized by Macro-Pinocytosis

**Figure 11:**
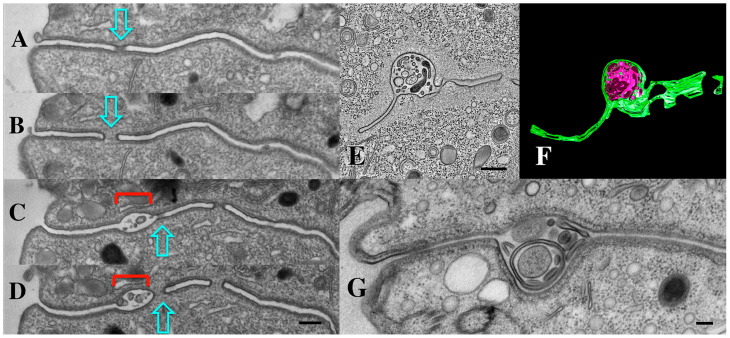
(**A**–**D**) Serial TEM section through a pocket of shed vesicles (jEMVs, red bracket) showing that it is flanked by developing conjugal fusion pores (blue arrows), (Scale bar = 250 nm). (**E**,**F**) Tomogram of *bcd1* × *bcd1* mating junction showing presence of junction pores, and over-abundance of jEMVs. Scale bar = 500 nm. (**G**) TEM image of mating junction in *HAP2Δ* × *HAP2Δ* mutant pair (Modified from Pinello, et al. [37]). Note absence of junction pores and hypertrophied, multi-membrane shed vesicles (jEMVs). Scale bar = 100 nm.

As mentioned earlier, we first observed extracellular micro-vesicles (EMVs) being shed within the narrow extracellular space that separates cells engaged in conjugation (Figure 11) (see also [34]). These ‘junction EMVs’ (jEMVs) appear in close proximity to the expanding membrane pores. Serial sections reveal that even when a single EM section suggests that jEMVs were being shed into a space with no neighboring pores (Figure 11C), such pores always appeared either above or below the plane of that original cross-section (Figure 11A–D).

### 3.8. Endocytosis and Membrane Fusion Influence Vesicle Shedding at the Mating Junction

TEM profiles suggest that junction vesicles (jEMVs) are formed through membrane budding directly from the plasma membrane and into the extracellular space of the mating junction (Figure 11C,D). This supports the interpretation that jEMVs represent a form of ‘ectosome’ rather than ‘exosomes’. As demonstrated earlier, when cells are compromised in endocytosis (as seen in the *bcd1* mutant), vesicles shed into the mating junction appear over-abundant (Figure 11E,F). This suggests that jEMVs accumulate within the junction as a result of reduced endocytic retrieval. Matings between cells that lack the conserved gamete-fusogen, *HAP2Δ*, also exhibit interesting and unusual features (Figure 11G) (see [31]). In the absence of the fusogen, pores fail to form, and consequently, the mating junction is devoid of cytoplasmic bridges or pores (this prevents nuclear exchange resulting in a form of self-fertilization or autogamy). Second, though EMVs appear to be extruded into the mating junction, they appear grossly abnormal. The jEMVs of (*HAP2Δ* × *HAP2Δ*) pairs are enlarged. jEMVs were measured from our entire collection of micrographs for both wild-type and mutant matings. The results appear in Figure 6B. EMVs photographed in situ show a dramatic doubling in size within the *HAP2Δ* × *HAP2Δ* junctions. Wild-type junctions exhibit an average jEMV diameter of 56.7 nm (std. dev 26) vs. 119.7 nm (std dev 71) for jEMVs observed within *HAP2Δ* junctions. Ciliary EMVs (cEMVs) harvested from wild-type and *HAP2Δ* cells by centrifugation show almost no difference in EMV diameter (Figure 6A, avg = 105 nm for wild types, std = 38, and avg = 124 nm for *HAP2Δ* pairs, std = 50).

We suspect that this is because centrifugation collects predominantly vesicles shed by cilia during co-stimulation (as jEMVs are trapped withing the mating junction cleft), and ciliary EMVs (cEMVs) are produced in a fashion independent of *HAP2* gene expression. These observations suggest that membrane fusion mediated by the Hap2 gene product is involved not only in pore formation, but in jEMV production and/or processing. This involvement remains mysterious, but one possibility involves a potential role for the Hap2 fusogen in completing macro-pinocytotic retrieval. A membrane ruffle forms and engulfs a collection of vesicles, but is unable to fuse, thereby completing the process.

## 4. Discussion

### 4.1. Membrane Vesicle Shedding Occurs in Ciliates

There are two previous reports of membrane shedding from *Tetrahymena.* Dentler [58] was the first to identify vesicles being shed from the cilia of *Tetrahymena* under natural growth conditions. Another report is from a rather unusual experiment. Ciliates, both free-living and parasitic, express an abundance of surface antigen proteins [59,60,61]. Antibodies can immobilize the cells, presumably by cross-linking surface antigens expressed on the cilia, hence the name ‘immobilization antigens’ or i-antigens [62,63]. Bisharyan et al. [64] engineered the expression of a foreign surface-antigen from the parasitic ciliate *Ichthyophtherius* (‘Ich’) in the free-living ciliate *Tetrahymena* [64,65]. Antibodies for the Ich antigen were applied to these transgenic *Tetrahymena* cells. Several responses occurred in quick succession. First, there was a transient, dramatic rise in intracellular calcium followed by vast shedding of 50–100 nm membrane-bound tubules and vesicles loaded with ‘Ich’ surface antigens. Although this is a highly exotic laboratory situation, it raises the possibility that *Tetrahymena* could shed their own endogenous surface antigens through micro-vesicles under normal life-history conditions, a possibility first raised by Ko and Thompson [66].

Our investigation confirms that there are in fact multiple types of vesicles shed from surface membranes in *Tetrahymena* as a part of their life history. We present evidence suggesting that 100 nm ectosome vesicles shed from cilia during co-stimulation participate in the process of co-stimulation. It is noteworthy that these naturally shed micro-vesicles carry mating-type proteins and the SerH3 surface antigen. Second, we observed smaller 60 nm vesicles (jEMVs) shed from the ‘naked’ (un-ciliated) surfaces that form the mating junction during conjugation.

### 4.2. Shed Micro-Vesicle Contents from Tetrahymena Resemble Micro-Vesicle Proteomes from Other (Distantly Related) Phyla

The proteome of our 4 h EMVs resembles that of shed vesicles analyzed from other systems including evolutionarily distant phyla. Vesicles shed from a *Drosophila* hemocyte cell line produce a proteome that is strikingly similar to ours [50]. Of the 100 most frequently identified mammalian proteins harvested from extracellular micro-vesicles, 30 match proteins were identified in our study [51,52].Two proteins that have become bio-markers for micro-vesicle identification, the 14-3-3 protein and members of the tetraspanin protein family, also appear in our EMV proteome.

Among protists, most work characterizing extracellular micro-vesicles has been carried out on parasitic genera such as *Trichomonas*, *Plasmodium*, *Toxoplasma*, *Giardia*, *Trypansomes and Leishmania* (reviewed by [67,68]. *Tetrahymena* cEMV proteins closely resemble the proteome of micro-vesicles published for the parasitic flagellate *Trichomonas vaginalis* [69,70]. Among the more closely related alveolate parasites, the most thorough proteome at the time of writing is from *Plasmodium* [71]. Here, the micro-vesicle proteome resembles that of *Tetrahymena*, and interestingly, the ultrastructure of the *Plasmodium* micro-vesicles is also similar (disk-shaped structures appear in TEM profiles). In parasitic protists, micro-vesicles carrying both protein and RNA cargo have been linked to elevated cell adhesion (parasite to host cells), and modification of the host tissue environment [67,70,72]). Very few studies have explored extracellular micro-vesicles in free-living protists. Among those few, ref. [58] identified vesicles being shed from the cilia of *Tetrahymena*, but it is in the flagellated green algae, *Chlamydomonas*, that we find the closest parallel to our own study [23,73]. ‘Plus’ (male) *Chlamydomonas* cells secrete vesicles following contact with ‘minus’ (female) cells. These vesicles are enriched in a specific protein associated with mating and are capable of triggering a mating response in minus cells upon binding with their flagella. Among these responses is the up-regulated expression of the gamete-specific fusogen, Hap2 [23], also dramatically up-regulated following co-stimulation in *Tetrahymena* [31].

### 4.3. Proteins Involved in ‘Ectosome’ Formation Are Present in the Tetrahymena EMV Proteome

Ectosome shedding (direct plasma membrane budding) has been richly characterized in other model organisms. From such studies, we learn that ectosome shedding can be stimulated by elevating intracellular calcium [52]. Elevated intracellular calcium levels support ectosome budding in a variety of ways. Ca^++^ can induce scramblase and floppase activities while inhibiting flippase lipid transporters. This causes disruption of the asymmetric lipid distribution between inner and outer leaflets. Phosphatidyl-serine (normally restricted to the inner cytoplasmic leaf of the phospholipid bilayer) becomes visible on the outer leaf where it contributes to the membrane curvature that supports vesicle budding [74,75]. It is significant that our cEMVs contain multiple calcium-transporting ATPases and calcium-binding proteins, in addition to one scramblase and three floppase candidates. These ‘floppases’ were originally annotated as flippases in the Tetrahymena genome database, i.e., proteins that direct lipids from the inner leaflet to the outer leaflet, but upon further analysis, it is likely that these represent ‘floppases’ directing lipids from the extracellular to intracellular leaflet (Naomi Stover, personal communication).

Intracellular Ca^++^ elevation can lead to degradation of the cortical actin cytoskeleton in a way that promotes ectosome release. Ca^++^ activates calpain proteases that cleave actin-capping proteins leading to disruption of the connection between the cortical actin cytoskeleton and the membrane [76]. This disrupted connectivity between the surface membrane and the actin cytoskeleton is hypothesized to encourage membranes to undergo budding [77]. It is notable that actin and actin-capping proteins both appear in our EMV proteome, as well as a number of calpain-family proteases.

Once membrane budding has been initiated, another function, that of vesicle abscission, is mediated by ESCRT-III polymer formation and AAA-ATPase activity [78]. Originally associated with MVB formation and ‘exosome’ production, ESCRT-III-mediated micro-vesicle production have also been linked to ‘ectosome’ production [78]. ESCRT-III proteins form polymers that coil, constricting the neck of budding membranes whether projecting into the lumen of a multi-vesicular body or into an extracellular space. Snf7 is one of the most abundant ESCRT-III subunits responsible for this coiling ligature. The final act of abscission requires a V-type ATPase [79]. *Tetrahymena* homologs to Snf7 were present in our EMV proteome, as were multiple subunits of vacuolar-type AAA ATPases.

### 4.4. A Role for the Proteasome

The third most abundant class of proteins in our shed micro-vesicles were related to protein ubiquitination and proteasome degradation. Proteasomes have historically been associated with an intracellular context. However, studies have found that functional proteasomes can be expressed in the extracellular environment where they can mediate protein degradation. Lai et al. [80] identified proteasomes co-purifying with exosomes in circulating blood serum in mammals. Bochmann et al. [81] demonstrated that functional proteasomes are exported from activated immune cells into the circulating blood serum by way of ‘microparticles’, the dissolution of which may lead to the generation of active extracellular proteasomes. A number of roles have been proposed for extracellular proteasome activity including roles during metazoan fertilization. Zimmerman et al. [82] report that extracellular proteasomes (from acrosome exocytosis) might represent the ‘lysins’ historically associated with penetration of the egg zona pelucida. Sawada et al. in [83] linked extracellular proteasome activity of micro-vesicles to proteolytic degradation associated with fertilization in ascidians (See also [84]). The collective profile of proteasome components within *Tetrahymena* vesicles raises the following interesting question: could there be a role for extracellular proteolysis during conjugation?

### 4.5. Membrane Vesicles Trigger Pairing in Ciliates

Ciliary membrane material has been associated with mating activity in both *Tetrahymena* and *Paramecium*. In *Paramecium*, it was demonstrated that dead cells of one mating type can trigger self-pairing in cells of the complementary mating type [85,86], reviewed by Metz [87], and Hiwatashi [88]. These induced homotypic pairs undergo normal nuclear events associated with conjugation, i.e., meiosis and nuclear reorganization, resulting in a novel form of self-fertilization. Subsequently, it was shown that cilia isolated from one mating type could trigger homotypic pairing in cells of the complementary mating type [89,90]. Membrane vesicles mechanically generated from disrupted ciliary preparations were shown to trigger homotypic pairing in cells expressing the complementary mating type [91,92,93]. Finally, it is significant to note that a ciliary protein was recently identified that may serve as the mating type protein in *Paramecium* [26].

Attempts to repeat these experiments in *Tetrahymena* failed to produce a similar result [94]. Dead cells do not provoke self-mating in *Tetrahymena* as seen in *Paramecium*. Ciliary membrane vesicles have been generated artificially from *Tetrahymena* (as in the *Paramecium* experiments). Cilia were sheared by calcium shock and disrupted mechanically by vortexing. The resultant membrane–vesicle fraction was pooled by centrifugation and assayed for mating activity [95]. These mechanically generated ciliary membrane vesicles do seem to elevate the percentage of cells engaged in mating when applied to a mixed-mating-type starvation culture.

Our study is the first to demonstrate that endogenous membrane-bound materials (cEMVs) are shed naturally and contribute to the cell signaling that results in co-stimulation and pair formation in *Tetrahymena*. When co-stimulated cells are removed from conditioned media, pairing is delayed until (presumably) the cells are able to secrete cEMVs in sufficient abundance to support cell signaling and cell adhesion. Supplementing the un-conditioned medium with purified cEMVs restores mating activity to the rate seen in conditioned medium. Supplying excess cEMVs to already conditioned media can actually accelerate pairing kinetics. All these results suggest that ciliary EMVs shed from co-stimulating *Tetrahymena* possess a mating activity that is necessary for the cell–cell signaling that triggers conjugation.

As mentioned earlier, there is a precedent for this type of cell signaling. *Chlamydomonas* shed vesicles from their flagella in response to ciliary adhesion and these appear to mediate cell signaling associated with mating [23,73]. Shed vesicles collected from mating *Chlamydomonas* plus cells are capable of triggering expression of the conserved gamete specific fusogen, Hap2, in minus cells. It is noteworthy that both *Chlamydomonas* and *Tetrahymena*, among a growing gallery of organisms from widely divergent kingdoms, all deploy this same *HAP2* homolog as a gamete-specific fusogen [31,39].

### 4.6. Extracellular Micro-Vesicles Shed from Tetrahymena Cilia (cEMVs) Resemble the ‘Factor Activate in Conjugation’ (FAC) Described by Jason Wolfe

Forty-five years ago, Adair and Wolfe demonstrated the need for an extracellular substance secreted into the surrounding medium that supports both co-stimulation and cell adhesion [13,17,19]. This factor is heat-stable, retained by dialysis (suggesting that it was not a small molecule), and could be collected from either starving or co-stimulating cells. We repeated the Wolfe lab experiments, confirming that co-stimulated cells secrete a substance into the surrounding medium that is necessary for mating (Figure 5). We then demonstrated that EMVs, harvested by differential centrifugation, possess all the characteristics exhibited by Wolfe’s ‘factor’.

Wolfe’s ‘factor active in conjugation’ (FAC) had the following properties: it was necessary for mating (washing cells blocks or delays pairing; adding back conditioned medium restores it); the FAC was large (retained by dialysis); heat stable (up to 30 min in boiling water); and it could be produced by either mating type (it lacks mating-type specificity). We observed many of these characteristics in our ciliary EMVs. They are large (100 nm); stable after immersion in boiling water; and both necessary and sufficient for supporting co-stimulation and cell adhesion (washing cells blocks or delays pairing; adding purified cEMVs to washed cells restores pairing dynamics or even accelerates it). Pre-incubating cells of one mating type with cEMVs from co-stimulating cells does not reduce the co-stimulation period, but adding cEMVs at the time of cell-mixing does. All these observations suggest that we have identified Wolfe’s ‘factor active in conjugation’.

### 4.7. cEMVs Serve as Mobile Platforms, Expanding Surface Presentation of MT Proteins During Brush-By Encounters

The mating-type gene locus encodes two proteins for each individual mating type, (MTA and MTB). Yan et al. [27] recently performed knockouts of the individual mating-type alleles (MTA and MTB) and analyzed the impact on co-stimulation. They concluded that the two mating-type proteins function as a complex within each partner. Using immuno-precipitation and HA tagging, they pulled down a suite of six other proteins (MRC1-6) that, with MTA and MTB, comprise a mating-type recognition complex (MTRC). Among the MRC subunits are K-Type ATPase proteins (TPA9) that appear to act as calcium translocators [96,97]. Our EMV proteome analysis revealed mating-type proteins and a K-Type ATPase as well (TTHERM_00923170).

Yan et al. [27] demonstrated localization of MT proteins on the plasma membrane, but not on ciliary membranes, an observation at odds with our hypothesis suggesting that cilia shed vesicles containing MT proteins. This contradiction may be understood in light of work from *Chlamydomonas*. Cao et al. [23] identified the algal mating factor (SAG1-C65) on EMVs that are shed from cilia, and also noted that these are absent from (or only weakly present in) the ciliary proteome. Numerous other proteins associated with ciliary vesicles were present in the plasma membrane but de-enriched in the cilia. These authors hypothesize that anterograde transport to the ciliary tips/EMVs is greatly accelerated during co-stimulation. They attribute this to fast transport of the mating factors by IFT (intra-flagellar transport), with short occupancy time in the cilium through which they travel. This could also be true of *Tetrahymena*. Of potential relevance, IFT52 was identified in our cEMV proteome.

### 4.8. Tetrahymena Conjugation Requires Active Endocytosis

**Figure 12:**
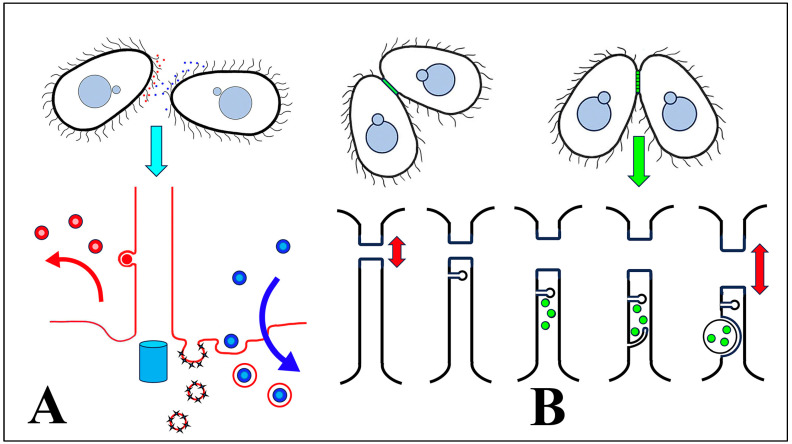
Summary of shed vesicle activity during *Tetrahymena* conjugation. (**A**) cEMVs (ciliary EMVs) are shed from cilia of cells expressing complementary mating types (indicated by red and blue) and transferred between cells during ‘brush-by encounters’. Lower diagram highlights red vesicles being shed from the cilium, and blue vesicles (from the mating partner) being internalized by endocytosis at the ciliary pocket and near sites of pinocytosis (the parasomal sacs). The turquoise cylinder represents the basal body subtending the cilium. (**B**) After pairs have joined, jEMVs (junction EMVs) are shed into the intercellular space within the mating junction (green). The lower diagram illustrates consecutive views of the mating junction over time. jEMVs (green) appear to be internalized by macro-pinocytosis, likely for degradation by autophagy. Red double arrows suggest pore expansion coincident with jEMV shedding and internalization (membrane excavation).

We hypothesize that cEMVs are shed and transferred from one mating partner to another during ‘brush-by’ encounters (Figure 12A). We further propose that extracellular vesicles from one mating type are produced as ectosomes budding from the ciliary tip, and then transferred to a cell of a complementary mating type where they become concentrated in the ciliary pocket. Two ‘pockets’ have been classically identified at the base of somatic cilia in *Tetrahymena*: a broad depression (the ciliary pocket) that deforms the plasma membrane all around the shaft of each cilium, and a specialized pit (the parasomal sac), localized anterior to each cilium. The latter serves as sites of active pinocytosis [98].

Observations from archival TEM images (Figure 9A) and from *bcd*1 mutants at the time of co-stimulation (Figure 10) convince us that the ciliary pocket is also involved in bulk transport of extracellular materials such as cEMVs. It is unclear whether this represents an unrecognized virtuosity of the parasomal sac, or if the ciliary pit in general engages in receptor-mediated endocytosis or macro-pinocytosis that is disabled by the dynamin inhibitor (dynasore) and in the *bcd*1 mutant. Significantly, others have demonstrated that pair formation is blocked by inhibitors of receptor-mediated endocytosis [99].

### 4.9. A Potential Role for sRNA in cEMV Signaling

A great deal of attention has been paid to the miRNA contents of shed vesicles, and their potential role in cell signaling and miRNA-mediated regulation of gene expression. Shed micro-vesicles collected from *Tetrahymena* during co-stimulation and later during mating (meiosis) are rich in RNA including small RNAs, some of which resemble sRNAs (in size, at least) previously characterized for *Tetrahymena* [56,57]. Micro-vesicles collected during co-stimulation exhibited two minor peaks of small RNA. One peak of small RNA corresponds to sequences ~24–25 nt in length. Lee and Collins [57] describe a similar class of sRNAs, ~23–24-nt, that may serve as guides for RNA cleavage, demonstrating a form of post-transcriptional gene silencing (PTGS). RNA analysis of jEMVs isolated from pairs undergoing meiosis revealed short RNA species that were mapped to 24,432 genes in the *Tetrahymena* genome.

The role of sRNAs in EMV activities is purely conjectural at this point. The least interesting possibility is that during cEMV harvest by differential centrifugation, intact cells are disrupted and RNAs are collected as cytoplasmic contaminants. We would argue that this is unlikely for two reasons: first, the initial centrifugations to eliminate intact cells are very gentle, involving a desk-top centrifuge and low-speed pelleting. Second, the final high-G centrifugations are not fast enough to pull down isolated small RNA molecules. The RNA detected must be associated with larger particles subject to the G-forces at play during their collection. Another uninteresting possibility is that, as EMVs form, they simply sample the neighboring cytoplasm during their formation. In that case, sRNAs detected in our EMV samples are a kind of accidental payload. We present a hypothetical possibility suggesting that RNA-laden cEMV vesicles play a more interesting role.

Hypothetically, shed micro-vesicles might trigger changes in gene expression associated with co-stimulation. cEMVs (with their sRNA cargo) could make contact with mating-type receptors during co-stimulation, bind, and trigger macro-pinocytosis internalizing the vesicles within the recipient cell. Subsequent fusion of the EMV membrane with the limiting endosome membrane would release the EMV contents into the cytoplasm where they could exert their action. miRNAs typically act by inhibiting gene expression. We envision that un-identified conjugal repressors may be active in starved cells, and that EMV-delivered small RNAs trigger conjugation by silencing these conjugal repressors. This scenario is modestly supported by the appearance of a Piwi-family argonaute homolog: Twi2 in our EMV proteome. Twi2 has been shown to be associated with 23–24-nt small RNAs [100], and has been implicated in PTGS [101].

### 4.10. A Possible Role for jEMVs During Membrane Remodeling

Non-ciliary EMVs appear to be shed directly from the plasma membrane and into the narrow extracellular space of the mating junction. jEMVs appear during a time of active membrane remodeling as Hap2-mediated fusion foci expand into larger pores, creating a weakened ‘membrane curtain’ through which gamete pronuclei are exchanged. We propose that jEMVs may serve to excavate membrane from neighboring pores, aiding in their expansion (Figure 12B) [34]. As the membrane is pulled from the margins of these expanding intercellular pores, it may be packaged as jEMVs within the inter-cellular lumen. From here, we observe what appears to be macro-pinocytosis, internalizing these shed vesicles. The process of jEMV shedding and internalization appears to require the gamete fusogen Hap2, without which such vesicles expand into enlarged multi-laminate vesicles without producing a pore. Normal jEMV processing also requires the Bcd1 protein, a protein with PKA-anchor homology, necessary for endocytosis [20]. Without wild-type Bcd1 activity, jEMV vesicles of normal size proliferate within the mating junction. It is notable that *bcd1* × *bcd1* pairs also fail to exchange pronuclei or complete normal conjugal development [102].

### 4.11. A Possible Role for jEMVs in scnRNA Elimination

Vesicles harvested from fragile *HAP2Δ* pairs four hours into conjugation (during meiosis II) produced more RNA than we expected. Sequence analysis revealed hits on 24,432 genes in the *Tetrahymena* genome (virtually the entire genome). This astonishing result makes sense when one realizes that during meiosis (at the time of the 4 h EMV harvest), *Tetrahymena* conjugants are busy broadcasting a rich collection of ‘scan’ scnRNAs that cover much of the micronuclear genome. These scnRNA species are used to interrogate the macronuclear genome in a complex process theorized to execute a form of germline surveillance [103,104]. In brief, micronuclear (germline) scnRNAs appear to be subtracted against macronuclear (somatic) RNAs, leaving mic-specific sequences that can guide chromatin remodeling. This process offers a pathway by which cells can prevent foreign DNAs that have successfully invaded the germline micronucleus from entering the somatic macronucleus.

It has not yet been described how the mic/mac RNA duplexes that are generated during genome scanning are subsequently eliminated. jEMVs (containing genome-wide RNA sequences) are extruded into the mating junction. From there, they are taken up via macro-pinocytosis and likely degraded via autophagy [34]. It occurs to us that jEMVs might not only help excavate membranes as junction pores expand, but they could represent a pathway by which dsRNA complexes are eliminated during meiosis-driven genome scanning. jEMV extrusion and uptake could play a role in scnRNA elimination. We cannot at this time distinguish between the ‘accidental payload’ scenario, and one involving directed sequestration of RNA within the jEMV payload.

## Data Availability

The original contributions presented in this study are included in the article/Supplementary Materials. Further inquiries can be directed to the corresponding author.

## Supplementary Materials

The following supporting information can be downloaded at https://www.mdpi.com/article/10.3390/microorganisms13040803/s1. Supplementary File S1: *Tetrahymena c*EMV isolation protocol; Supplementary File S2: cEMV Proteome.
