# Supplementary material for: The Role of Membrane-Bound Extracellular Vesicles During Co-Stimulation and Conjugation in the Ciliate Tetrahymena thermophila
Source: Microorganisms. 2025 Apr 1;13(4):803. doi: 10.3390/microorganisms13040803 (PMC12029339; doi:10.3390/microorganisms13040803)
Supplement: Supplementary file 1 [file microorganisms-13-00803-s001.zip › Supplementary File # 1 (EMV Cnj).docx]

Supplementary Materials

S1. EMV Isolation Protocol:

We inoculated six, 250 flasks with each complementary mating type (30mL of Neff media) and incubated overnight. Cultures were decanted into 6 Fernbach flasks (500mL Neff media each), and incubated overnight until cell density reached 300-400K/mL on a shaking incubator. Cells were then starved in surface-sterilized nunc plates at 1,000K/mL after washing in 10mM Tris media (pH 7.4) twice. To wash, we centrifuged cells in 50mL centrifuge tubes for 5 min at 25°C and replaced the supernatant with 10mM Tris media (pH 7.4) without disturbing pelleted cells. Cells were resuspended and washed once more to remove leftover Neff media from solution. Starving cells were placed in the 30C incubator overnight. Once starved, cells were consolidated by mating type, and equal numbers of cells mixed for each mating type to initiate co-stimulation. After 40 minutes of co-stimulation, we transfered cells into 50mL centrifuge tubes and began centrifugation at 300*g for 5 min at 25°C. Without disturbing pelleted cells, we transfered supernatant to new 50mL tubes and centrifuged again at 300*g for 5 min at 25°C. Once media from starved cells had been centrifuged twice, we transferred it to a container and kept cold without freezing (4°C). We prepared SW-32 and MLS-50 rotors (4°C) for ultracentrifugation. Centrifuged media was poured into SW-32 centrifuge tubes (Ultra-Clear Tubes 25 x 89 mm, Beckman Coulter) and centrifuged at 2,000*g for 30 min at 4°C to remove dead cells. Supernatant was collected and kept cold. Once all media was centrifuged, we performed centrifugation using SW-32 centrifuge tubes at 10,000*g for 50 min at 4°C. We then collected the supernatant and kept it cold. Once all the media was centrifuged, we performed another centrifugation using SW-32 centrifuge tubes at 100,000*g for 70 min at 4°C. This time, we discarded the supernatant without disturbing pelleted EMVs. The pellet was collected using a pipette and transferred to a clean MLS-50 ultracentrifuge tube (Ultra-Clear Tubes 13x51 mm, Beckman Coulter). We performed ultracentrifugation on concentrated EMVs in MLS-50 tubes at 100,000*g for 70 min at 4°C. We then discarded the supernatant, and transferred the pellet material to a single MLS-50 centrifuge tube. EMVs were resuspended, and centrifuged at 100,000*g for 100 min at 4°C. We carefully discarded the supernatant and collected the pelleted EMVs, keeping them concentrated in cold storage on ice in a fridge, or at -80°C for long-term storage.
